# Food Environments and Their Influence on Food Choices: A Case Study in Informal Settlements in Nairobi, Kenya

**DOI:** 10.3390/nu14132571

**Published:** 2022-06-21

**Authors:** Shauna M. Downs, Elizabeth L. Fox, Vincent Mutuku, Zacharia Muindi, Tasneem Fatima, Irena Pavlovic, Sana Husain, Minna Sabbahi, Simon Kimenju, Selena Ahmed

**Affiliations:** 1 Department of Health Behavior, Society and Policy, Rutgers School of Public Health, Newark, NJ 07102, USA; tf283@sph.rutgers.edu (T.F.); irenampavlovic@gmail.com (I.P.); minnas789@gmail.com (M.S.); 2 Master of Public Health Program, College of Veterinary Medicine, Cornell University, Ithaca, NY 14853, USA; elf23@cornell.edu; 3 G-Thamini Youth Group, Nairobi, Kenya; vmmswahili@gmail.com; 4 Map Kibera, Kibera, Nairobi, Kenya; muindre@gmail.com; 5 Bloomberg School of Public Health, Johns Hopkins University, Baltimore, MD 21205, USA; sanahusain95@gmail.com; 6 Kula Vyema Centre of Food Economics, Nairobi, Kenya; skimenju@gmail.com; 7 Department of Health & Human Development, Montana State University, Bozeman, MT 59717, USA; selena.ahmed@montana.edu

**Keywords:** food environment, food choice, diet quality, informal settlements, Kenya

## Abstract

The food environments that people have access to shape their food choices. The purpose of this study was to use mixed methods to characterize the external food environment in informal settlements in Nairobi, Kenya and to examine the individual factors that influence the way in which women interact with those environments to make food choices. We used a combination of food environment assessments (vendor mapping, collection of food prices, food quality assessments) and five focus group discussions with women (*n* = 26) in four villages within two informal settlements in Nairobi (Mukuru and Kibera) to better understand the drivers of food choice. We found a large number (*n* = 1163) of vendors selling a variety of food within the settlements. The highest number of vendors were selling fruits and/or vegetables; however, there was limited diversity of fruits available. Animal-source foods were considered relatively expensive as compared to plant-based foods, including prepared fried snacks. We found that the way women interacted with their food environments was influenced by individual factors such as income, time, convenience, and preferences. Our findings suggest that interventions targeting both the external food environment as well as individual factors such as income will be necessary to support healthy diets among low-income populations living in informal settlements in Kenya.

## 1. Introduction

The vast majority of countries, including many low- and middle-income countries (LMICs), face burdens of multiple forms of malnutrition [1,2], with a rising prevalence of overweight/obesity and diet-related non-communicable diseases (NCDs) alongside persistent undernutrition [3,4,5]. In Kenya, the percent of underweight women has declined from 13.1% in 2000 to 9.7% in 2016 [6]. Meanwhile, the percent of overweight women has increased by 24% (from 31.7% to 39.2%) and obesity has increased by 40% over this same time period (10.6% in 2000 to 15.1% in 2016) [6]. This high prevalence of overweight and obesity is more pronounced in urban settings, particularly among urban poor living in informal settlements [7,8,9]. In informal settlements in Kenya, 43% of women are overweight or obese [10]. The shifting burdens of disease in Kenya and other LMICs can be attributed to the “nutrition transition”, where populations move from traditional diets high in fiber and micronutrients to more processed diets high in sugar, fat, and salt, as well as animal-source foods. These shifts in dietary patterns often take place alongside reduced physical activity levels as people migrate from rural to urban areas and adopt more sedentary livelihoods. Globalization, urbanization, and economic development are upstream drivers of the nutrition transition that have led to dramatic changes to the food environments that consumers interface with in LMICs [11].

The food environment is where consumers make decisions about which foods to acquire, purchase, and consume [12,13]. The external food environment encompasses the “availability, affordability, convenience, promotion and quality, and sustainability of foods and beverages in wild, cultivated, and built spaces that are influenced by the socio-cultural and political environment and ecosystems within which they are embedded” [12]. Individual factors such as income, knowledge, health, skills, mobility, time, etc. influence how consumers interact with the external environment to make food choices.

To identify, implement, and monitor context-specific interventions aimed at reducing the dual burdens of malnutrition, evidence is needed to characterize the food environments that consumers interact with, as well as how individual factors influence these interactions. This evidence will allow for the design of targeted interventions for promoting healthy consumption patterns within the socio-ecological contexts in which people live. However, much of the food environment literature has focused solely on high-income countries [14,15] and on specific dimensions (e.g., availability) of the food environment. Although there has been a growing amount of food environment research in LMICs [16,17,18,19], critical research gaps remain regarding the multitude of external food environment dimensions and individual factors that influence consumer behavior [12,20,21,22]. These knowledge gaps limit the ability to design comprehensive interventions that have the potential to lead to marked shifts in diets and disease burdens. In order to address these knowledge gaps, the aim of this study was to use mixed methods to characterize the external food environment in informal settlements in Nairobi, Kenya and to examine the individual factors that influence the way in which women interact with those environments to make food choices. The findings inform whether interventions and policy solutions aimed at improving food choice would be appropriate across different settlements within Nairobi.

## 2. Materials and Methods

We conducted a mixed-methods, cross-sectional study within four villages in two informal settlements in Nairobi, Kenya: Kibera (Olympic, Gatwekera, Kianda, Soweto West) which was established in 1910 and Mukuru (Railway, Wape Wape, Sisal, Vietnam) which was established in 1980 [23] in order to assess the similarities and differences across informal settlements in Nairobi. The settlements are characterized by inadequate access to safe water (fee for access and limited piped water), little or no sanitation (most often dependent on latrines serving many households), poor structural quality of housing, overcrowding, and insecure land tenure [24]. Within each of the settlements, the four villages with the highest density of vendors were purposively selected based on feedback from the community-based organizations that we worked with in each setting. We used the village boundaries as the area in which food environments were mapped.

We collected data to capture the external food environment using the following methods: (i) GIS mapping of the type of food vendors, (ii) vendor type and properties data, (iii) examination of the quality and diversity of fruits and vegetables, and (iv) price and food availability. We conducted focus group discussions, including participatory social mapping, with women to understand how they interface with their external food environment. The study team worked with Map Kibera, a community-based mapping organization, to collect data regarding the external food environment in both settlements. Focus group discussions were conducted in collaboration with community-based organizations in both Kibera (Mirror of Hope) and Mukuru (G-thamini).

All data were collected between March and July 2019. This research received ethics approval from the Rutgers University Institutional Review Board and the Amref Health Africa Ethics and Scientific Review Committee in Kenya.

### 2.1. Overview of Food Environments

#### 2.1.1. Mapping of Food Vendors

Local enumerators were trained by the research team to conduct all the external food environment data collection. We collected the GIS coordinates of all the food vendors/outlets in four villages within both slums, which represented the villages within each informal settlement that contained the most food outlets. Supplementary Table S1 provides a description (including photos) of the different vendor type classifications. There was one larger grocery store as well as larger wet markets on the outskirts of each of the informal settlements; however, they were not captured in the mapping given that they were outside of the boundaries of the settlements. We also collected information regarding the vendors’ infrastructure based on walling material (i.e., fully protected, partially protected, or not protected) and the level of convenience of the foods sold.

#### 2.1.2. Food Availability

For each of the vendors assessed in the food environment mapping, we also collected information about the type of foods they sold and whether they were a permanent or mobile vendor in order to characterize the food vendor type. When permission was granted by the vendor, we also captured photos of the foods sold (35% of all vendors). In the case of small restaurants or “hotels” in the settlements that permitted photos, the photo was taken of their menu (15% of all 108 restaurants). In order to examine the diversity of fruits and vegetables sold within the two settlements, we used the Produce Color Diversity (ProColor) tool, developed by Ahmed et al. [25] with all vendors that sold fresh produce in our sample and for which we had a photograph of their fruit and vegetable offerings. ProColor measures the diversity of fruits and vegetables within a given market based on color categories as a proxy to detect the presence of phytochemicals. Dietary phytochemicals are bioactive non-nutrient constituents of plant-based foods that support nutrition and health by mitigating the diet-related NCDs [26]. ProColor can be used as a proxy for examining gaps in dietary phytochemicals that may be present within the food environments with which consumers interface. The types of fresh vegetables in each of the following categories were recorded: (1) dark green, (2) other green, (3) red, (4) orange and yellow, (5) purple and blue, and (6) white; the types of fresh fruit in each of the following categories were recorded: (1) green, (2) red, (3) orange and yellow, (4) purple and blue, and (5) white. For fruits or vegetables with multiple different colors (e.g., flesh vs. skin), the color of the flesh was assessed. The enumerators recorded the information about ProColor diversity of fruits and vegetables in both slums. Herbs were not included in the analysis. For the vendors that provided permission for photos to be taken (35%), we used the photos taken of fresh produce to conduct the ProColor diversity assessment after the data collection was completed rather than in real time, given time constraints of enumerators in the field. We calculated both overall ProColor scores in each of the informal settlements and those at the village level, given that this is a better reflection of the total diversity of fruits and vegetables that people have access to in their immediate surroundings.

#### 2.1.3. Food Affordability

Within each of the four villages in each settlement, we collected price and produce quality data in a subset of purposively selected vendors. The vendors were selected based on their geographical location within each village to capture vendors from different locations within the village, given that we anticipated vendors working next to one another to have similar prices. We captured the prices of a list of 37 commonly consumed foods from three different vendors within each village. The list of 37 foods were identified based on the 10 food groups used in the minimum dietary diversity score for women [27]. We then asked our community partners to identify the most consumed foods within each of the food groups as well as other key foods that are commonly consumed but did not fit within one of the groups (e.g., soda, cooking oil, etc.). They also identified the typical units sold. The enumerators recorded the cost per unit and size or quantity of the unit typically sold at each vendor.

#### 2.1.4. Food Quality

We assessed food quality with the Produce Desirability (ProDes) tool [28] with three fruit and vegetables vendors within each village. These vendors were the same vendors used to capture food price data. The ProDes tool examined the desirability of fruits and vegetables based on five observational sensory measures: (1) overall desirability, (2) visual appeal, (3) touch and firmness, (4) aroma, and (5) size [28]. Two enumerators independently rated the five sensory measures of each of the fruit and vegetables on a scale from zero to six, with six being the most desirable and zero being the least desirable. We selected fruits and vegetables that were commonly consumed in the diets of our study population during the season in which we collected data, based on feedback from our local partners. These included: pineapples, oranges, bananas, and mangoes as the fruits and kale, tomatoes, cabbages, sweet potatoes, and onions as the vegetables.

#### 2.1.5. Convenience and Promotion

The level of convenience of the foods sold by vendors captured in the food environment mapping was determined based on the definition by Poti et al. [29] that categorized foods as: (1) requiring cooking and/or preparation; (2) ready-to-heat; and (3) ready-to-eat. We further categorized foods into ready-to-eat meals which included dishes with more than one food/food group (e.g., githeri (traditional Kenyan dish of maize and beans), rice and beans, etc.) and snacks which were solely one food/food group (e.g., french fries, chapati, mandazi (a fried dough), etc.). Ready-to-heat foods were not available within the settlements. We assessed the packaging and branding of foods sold by describing whether the majority of foods sold by the vendor contained: (1) clear package (no branding); (2) packaged (with branding); or (3) unpackaged (e.g., open, fresh, or wrapped in paper).

### 2.2. Focus Group Discussions

Three focus group discussions were conducted in Kibera and two in Mukuru to gain insight into how women (*n* = 26) living in the settlements interfaced with their food environments. We solely included women in our sample, given their roles and responsibilities as critical decision-makers in the context of food environments [30]. Moreover, we have found high levels of female-headed households in these settings [31]. With the assistance of our community partners in both settlements, we purposively selected female study participants who ranged in age. The average age of the focus group participants was 34 years (range 19–57 years). A semi-structured focus group guide that included questions related to food purchasing and consumption for themselves and their households, food quality and preferences, and how food environment dimensions and diets have changed over time was used to lead the discussions. Each of the focus groups began with the participants conducting social mapping of their food environments. Participatory social mapping is an ethnographic technique in which focus groups draw maps of their local community, in this case of their food environments [32]. Through the social mapping we were able to gain insight into the way in which women describe their own food environments. We did not prescribe boundaries for the mapping of women’s food environments. Rather, we allowed women to define their own food environments without any predefined boundaries. To conduct the social mapping, participants were given a large piece of white paper or a white board to map their food environment. They were asked to draw the features of their local food environment (e.g., the various places where they purchase food). After mapping the food outlets, they were asked what types of foods they purchase at each of the locations. The social mapping was followed by a discussion that explored experiences, perceptions, and priorities related to the food environment, including those related to the social map.

The focus groups (including the social mapping) were moderated by trained local partners with the assistance of a member of the research team. The focus groups were conducted in a combination of English and Swahili. All focus group discussions were recorded, translated into English (as needed), and transcribed verbatim to facilitate data analysis.

### 2.3. Analysis

The GIS coordinates of the food vendors were mapped using OpenStreetMap. The maps depicted the different types of vendors as well as the different foods they sold. Descriptive statistics and chi-squared tests were used to describe the properties of vendors and the average food prices across different types of foods using Microsoft Excel (Redmond, WA, USA) and SPSS (IBM Statistics, version 28, Armonk, NY, USA). We examined the photos of the menus of the restaurants to assess the proportion that sold fried snack foods (e.g., mandazi, french fries, etc.), fried meats (e.g., fried chicken), traditional meals (e.g., ugali (with or without vegetables, fish, etc.)), and roasted meats, as well tea and soda.

The total ProColor diversity score for fruits and vegetables was calculated by summing the items counted for fruits and for vegetables at each vendor, with a higher number denoting higher diversity. We also calculated the total color category diversity for fruits and for vegetables by summing the color categories where items were present (e.g., between 0 and 6 based on the different color categories) [25]. Total ProDesirability scores were calculated by averaging each of the five sensory parameters (i.e., overall desirability, visual appeal, touch and firmness, aroma, and size) for the fruits and vegetables examined. Means and standard deviations of total ProDesirability scores, as well as produce specific scores, were examined. We used independent t-tests to examine differences in ProDesirability scores across study settings. In addition, we calculated the inter-rater reliability (IRR) between enumerators’ ratings of produce using Cohen’s kappa statistic (see Supplementary Table S2). Based on the kappa statistic results, agreement between raters was fair to moderate [33]. Prior to data collection the enumerators discussed as a group the attributes of desirable versus less desirable fruits and vegetables to ensure that they were aligned with one another.

Focus group discussions were open-coded and codes were organized according to key themes related to the different dimensions of the food environment (availability, affordability, convenience, promotion, and quality) and the individual factors that influence how consumers interface with food environments. Given the mixed-methods nature of our data, we integrated the quantitative and qualitative data throughout the results to demonstrate triangulation of findings.

## 3. Results

The findings characterize multiple dimensions of the external food environment in informal settlements in Nairobi, Kenya and the factors that influence how women interfaced with them.

### 3.1. Overview of Food Environments

The vendors in both informal settlements were either permanent vendors (64% in Kibera; 61% in Mukuru) or street vendors selling food at fixed locations (36% in Kibera; 39% in Mukuru), with various degrees of infrastructure (see Supplementary Figure S1). All of the vendors captured in the mapping were a part of the informal or traditional food sector (e.g., independently owned kiosks, wholesalers, grocers, butchers, etc.). The social maps provided complementary information to the GIS maps, including the markets that were accessed beyond the informal settlements themselves. This allowed us to have a more complete picture of the ways in which women interact with their food environments.

### 3.2. Dimensions of the Food Environment

#### 3.2.1. Availability

**Figure 1:**
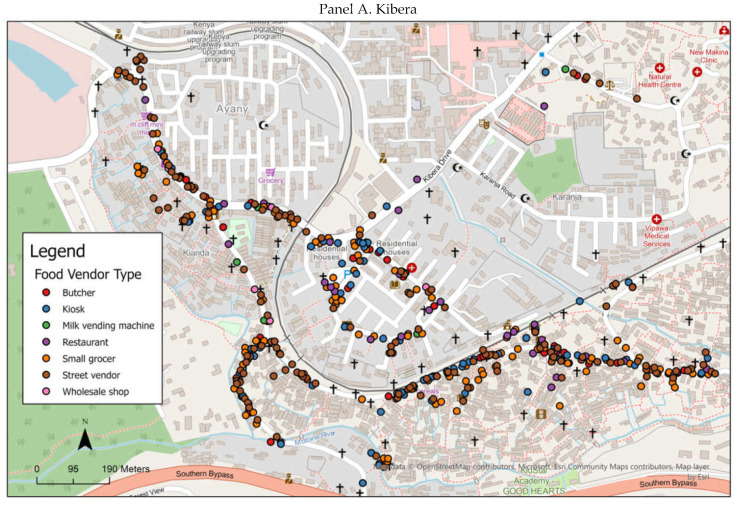

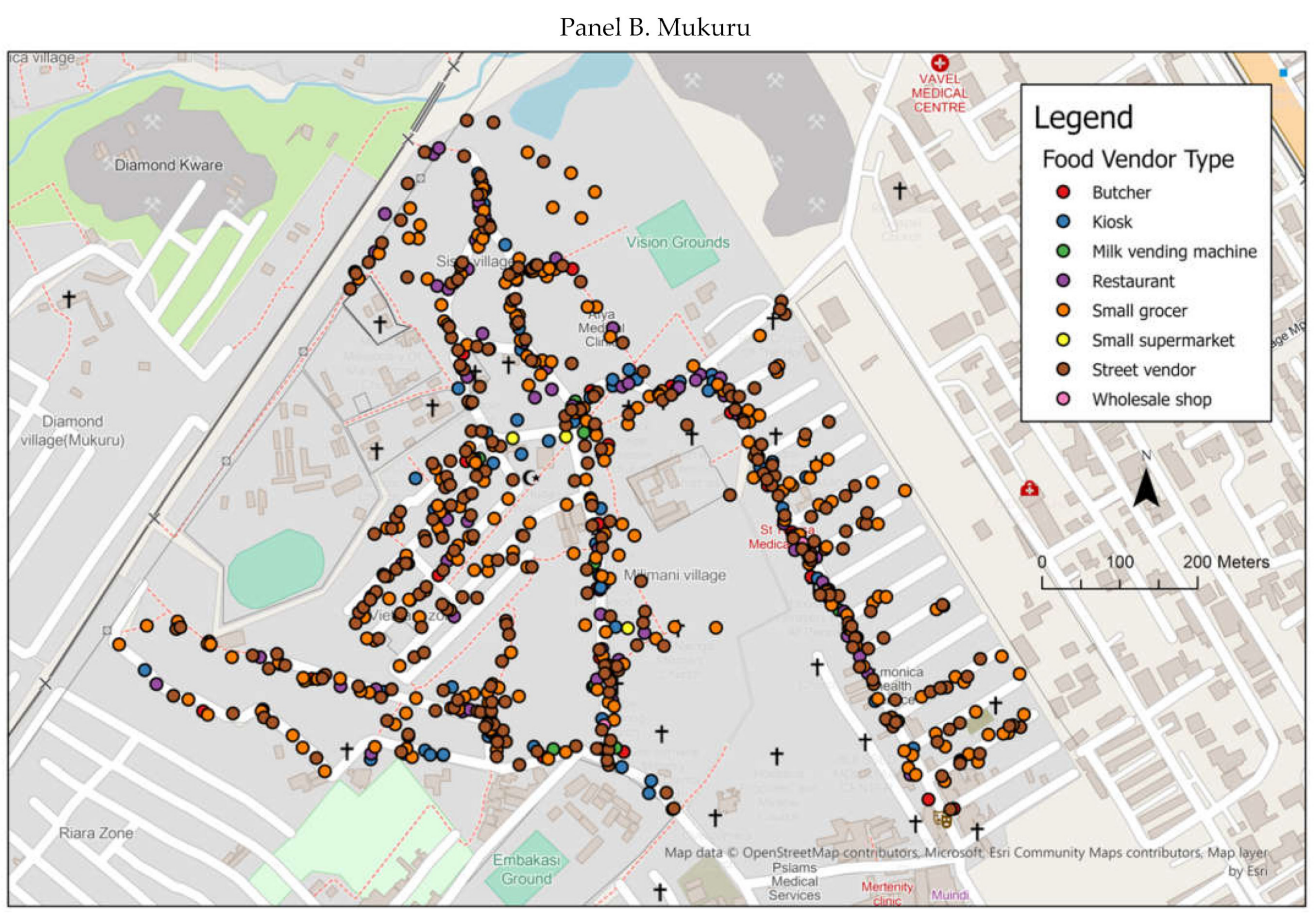
A map of the different types of food vendors in Kibera (Panel **A**) and Mukuru (Panel **B**).

**Figure 2:**
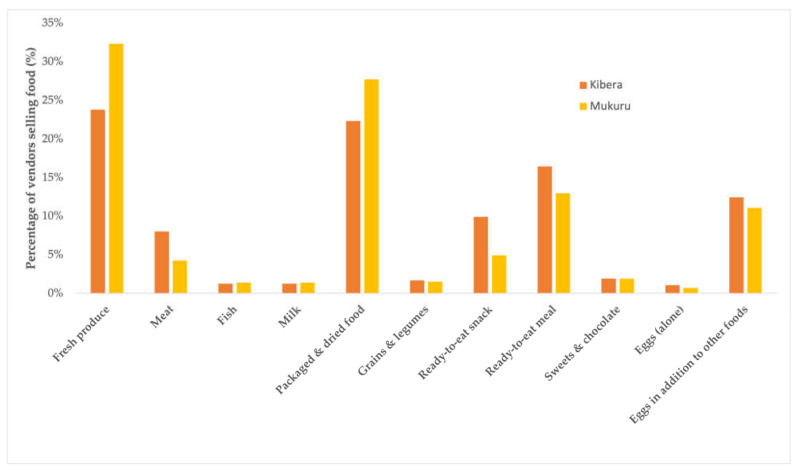
An overview of the types of foods sold by the vendors in the informal settlements.

Overall, we assessed 1163 vendors in the four villages in each of the informal settlements. Figure 1 depicts the maps of the different vendor types in the informal settlements. The majority of the food vendors were positioned along the roadside in both settlements. The most commonly sold foods were fruits and/or vegetables, followed by packaged and dried foods and ready-to-eat meals (Figure 2). A smaller number of vendors sold animal-source foods such as milk, meat, and fish. Although few vendors sold exclusively eggs, many sold eggs in addition to fresh produce or other types of foods. For this reason, they are presented in Figure 2 in both ways. Based on the photos of menus taken at a small number of restaurants within the settlements, the majority sold fried snack foods (63%), traditional meals (63%), and tea (56%). A smaller number sold fried meats (25%), roasted meats (19%), and sodas (19%).

In addition to examining the availability of vendors and foods sold, we also examined the diversity of fruits and vegetables sold by the vendors. Overall, the mean vegetable diversity score was 7.2 ± 3.2 in Mukuru and 7.5 ± 3.4 in Kibera, and there were no notable differences between the settings (*p* = 0.618). The fruit diversity scores were substantially lower than the vegetable scores. Overall, the mean fruit diversity score was 3.4 ± 1.6 in Mukuru and 4.2 ± 2.2 in Kibera, with significantly lower fruit diversity scores noted in Mukuru compared to Kibera (*p* = 0.027). Table 1 provides an overview of the ProColor diversity scores calculated at the village level in each setting. The highest vegetable diversity scores were for leafy green vegetables, whereas the highest fruit diversity scores were for orange and yellow fruits. 

In the focus groups, women described determining which food access points to purchase foods from based on the types of foods purchased or needed, as well as individual level factors that influenced participants’ ability to access these food access points. Overall, participants reported mainly accessing fruits and vegetables in either wet markets adjacent to, or kiosks within, the informal settlements. Fruits and vegetables purchased from wet markets were described as cheaper, but the distance to travel to the market was further, which was a barrier to purchase. Moreover, fruits and vegetables purchased from the wet market were sold in larger quantities which both increased the value for money but also the need for more resources to purchase the larger quantities. The ability to store the perishable foods was considered a disincentive for purchasing fruits and vegetables in larger quantities given that many women noted that their households lacked cold storage. Women reported that the main advantages of purchasing fruits and vegetables from kiosks rather than wet markets was the ability to purchase food in smaller quantities and with credit, as well as the added convenience given the larger distance to the wet markets. Moreover, pieces of fruit and some vegetables (e.g., pumpkin) were sold in the kiosks, which made it easier for women to purchase them given their income constraints. Most staple foods were purchased from kiosks in the settlements; however, participants indicated that they preferred to purchase them from chain supermarkets in the formal food environment given better variety, quality, and transparency. As one focus group participant said: “*For example we see rice meant for the market overstays in the shop without being sold. When you cook it,* [it] *tastes different from the supermarket one* (FGD M2)”.

Women described purchasing certain foods from specific vendor types and vendors due to concerns about hygiene, affordability, freshness and quality, and the source of the food (e.g., farmers selling directly to consumers at wet markets). Fish and meat were generally purchased from butchers or street vendors and processed foods from kiosks or supermarkets. Women discussed the informal quality checks that they typically do when making decisions about which vendors to purchase foods from, including examining how the vendor handles the food, examining the cleanliness of the kiosk, and smelling the food, as well as their past experience with a given vendor and whether they attributed the foods purchased from them to lead to sickness. Women described purchasing prepared meals and snack foods from restaurants or street vendors, particularly when children were at school and would be receiving a meal while there. This allowed women to cut down on cooking fuel in terms of preparing the foods themselves as well as water use for cleaning the dishes. It also saved them time, which was important given that most women worked in the informal sector and would try to earn money on a daily basis to cover food costs. Fewer participants indicated accessing food from the cultivated environment in rural areas. In these cases, they would source foods from family members who remained in the rural areas, given the perceived better transparency as compared to foods acquired in the informal settlements. One woman said: “*chicken are injected with chemicals so if you want some, you have to go home [to rural areas] or order from home so as to eat the traditional chicken*” (FGD1K).

In the focus group, participants indicated that the foods they most often consumed were available within the settlements. However, they noted changes in the availability of foods over time. They mentioned an increase in the availability of cakes, chicken, soda, biscuits, pilau, rice, and bread. They also noted greater availability of different species of fish (most imported from China) and varieties of fruit. However, women reported that the availability of foods and the quantity sold at a given price changed across seasons, which impacted where they accessed those foods at different times of the year. As one focus group participant said: “*Like during rainy season, there is plenty of food and during dry season, the food becomes scarce so you go far to buy food*” (FGDK1). Women reported that although food is more widely available and cheaper during the rainy season, there are also physical infrastructure challenges in terms of accessing these foods. As one focus group participant said: “*When it rains, the environment is not good and since the path we use is somewhere behind, you fear passing through there so you just buy from the kiosks right outside*” (FGDM1). As such, challenges in terms of accessing food were present and varied across seasons in participants’ food environments.

#### 3.2.2. Affordability

Supplementary Table S3 provides an overview of the prices of key foods from selected vendors in both informal settlements. Overall, there was little variability in the food prices across different vendors in the informal settlements. However, we did observe variability in the availability of specific foods, leading to fewer price observations for some foods and some variation across informal settlements (see Supplementary Figure S2). Staple and ready-to-eat foods such as french fries, chapati, mandanzi, etc. were relatively inexpensive (based on price data as well as focus group discussions) compared to nutrient-rich foods (e.g., eggs, fish, fruit, and some vegetables), which were more expensive.

Based on our focus group discussions, affordability was a key factor for where women purchased food and what they purchased. Participants indicated that higher costs were associated with greater quantities of food (e.g., inability to purchase in smaller units). They also highlighted the importance of purchasing food on credit, which they could do in kiosks but which they were unable to do from supermarkets in the formal built environment.

Women in the focus groups indicated that lower quality foods were sold at a lower price, leading women to weigh the trade-offs of higher quality foods with a higher price. In particular, women reported that in order to consume high-cost nutrient-rich foods such as fish and meat, they often purchased poor quality cuts of meat or fish with little flesh left on the bones as these were sold at a lower price. Although beans and maize were considered affordable to focus group participants, many fruits (e.g., apples, papaya, avocados, etc.), some vegetables (e.g., arrowroot, pumpkin, sweet potatoes, etc.), and animal-source foods were not. Women’s perceptions of the affordability of many of these foods were supported by the collected food price data. The lack of affordability of these foods impeded their ability to incorporate them into their diets. As one focus group participant explained: “*The most challenging issue is how to put your diet orderly especially when you do not have money. For example, today you get those things on credit then tomorrow you are not lucky to get a job so you still don’t have money, this will make to eat unbalanced diet because you [have] no choice*” (FGD1K).

#### 3.2.3. Convenience and Promotion

Overall, we found that most vendors in both settlements predominantly sold food items that required cooking and/or preparation (70% in Kibera and 75% in Mukuru), with much fewer selling ready-to-eat snacks or meals. Although the majority of food items were unpackaged or wrapped in paper in both informal settlements (95% in Kibera and 88% in Mukuru), with few foods being sold in clear packaging and/or branded packaging, Mukuru had a significantly higher proportion of branded products being sold by vendors compared to Kibera (1% in Kibera; 10% in Mukuru; *p* < 0.001). There were no other significant differences between the two informal settlements. Women indicated in the focus group discussions that packaged, ultra-processed foods were not commonly consumed, even if available within the settlements, given income constraints. As one woman stated: “*if it is the woman’s responsibility to provide everything, things like soda and biscuits are uncommon. The mother will hustle to get flour and vegetables for the children to eat and get satisfied, but for biscuits and sodas, you just tell them to wait for another day*” (FGDM1).

Based on the focus group discussions, women weighed convenience of purchasing foods from street vendors against the cost of cooking oil, the time to prepare foods, and access to water for washing dishes while cooking foods at home. As one focus group participant said: “*With 20 shillings, you eat at the hotel and save the kerosene to cook with in the evening*” (FGDM1). However, this depended on the amount that needed to be cooked. Some participants indicated that the higher cost of purchasing foods from street vendors was not worth the convenience factor if cooking for the whole family: “*Because, if I buy that 1Kg of wheat flour, I can make chapattis for the whole family and if I go buying from the hotel, no*” (FGD2K).

In terms of the promotion and branding of foods, focus group participants indicated that they were exposed to advertisements through television and movies, signage outside of hotels, outside supermarkets, and on roadsides, as well as advertisements in newspapers. The foods that were most often advertised included: cakes, chips, spaghetti, instant noodles, kebabs, eggs, meats, fish, pizza, and pilau. With the exception of instant noodles, none of the women in the focus groups mentioned specific brands when discussing food promotions. Women indicated a preference for trying the foods included in some of these advertisements, but the lack of affordability impeded their ability to purchase many of those foods. As one participant said: “*I do not have money for pizza and I just see people buying, hey [all laughs] I am really longing to eat pizza*” (FGD2K).

#### 3.2.4. Food Quality

**Figure 3:**
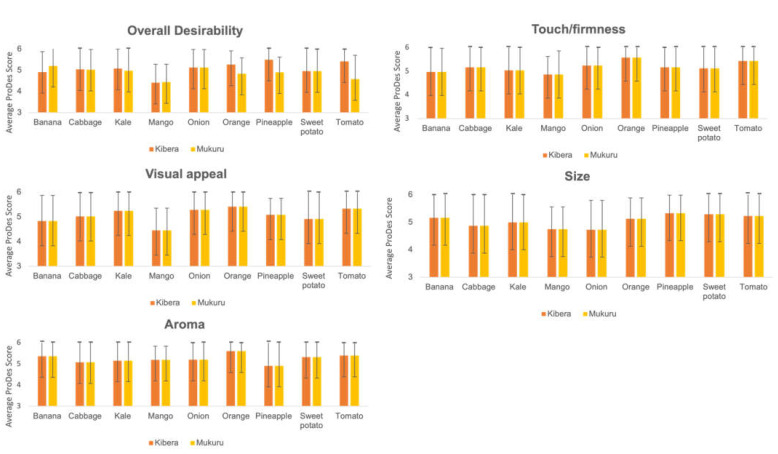
An overview of the ProDesirability scores for key fruits and vegetables in Kibera and Mukuru.

We examined the quality of fruits and vegetables being sold within the slums using the ProDesirability tool. Figure 3 and Supplementary Table S4 provide an overview of the sensory ratings for key fruits and vegetables in both informal settlements. We found more favorable ratings of the overall desirability (*p* = 0.011), size (*p* = 0.008), and aroma (*p* = 0.01) properties of fruits and vegetables in Kibera as compared to Mukuru. The overall desirability of fruits and vegetables was high, with little variability across settings and fruit and vegetable types. Pineapples and onions had the highest scores, and mangoes had the lowest.

Focus group participants described many concerns regarding quality of foods available within the settlements, particularly as quality relates to food adulteration. Quality was often described as concerns related to the quality of edible oil and meat, and not those of fruits and vegetables. For instance, one participant said: “*The cooking oil that is used in frying fish and chicken is not good oil; they use transformer* (fluid from electrical transformers) *oil to fry the fish*” (FGD2K), whereas another said: “*You can go to buy meat, when you cook it, turns black instead of appearing like meat, so you can buy meat and then you fail to eat it*” (FGD2K). Women mentioned that there were rumors throughout the settlements about the quality of meat being sold, including that donkey was being sold as cow or goat meat. This made them less likely to purchase meat, in addition to its high cost. One woman described “*kienyeji chicken*”, which is free range chicken found in villages, being sold in the settlements but at a higher price (KES 450 vs. 700) than the typical chicken sold. In particular, women attributed the poor quality of oils and meat with cancer and other negative health outcomes.

**Figure 4:**
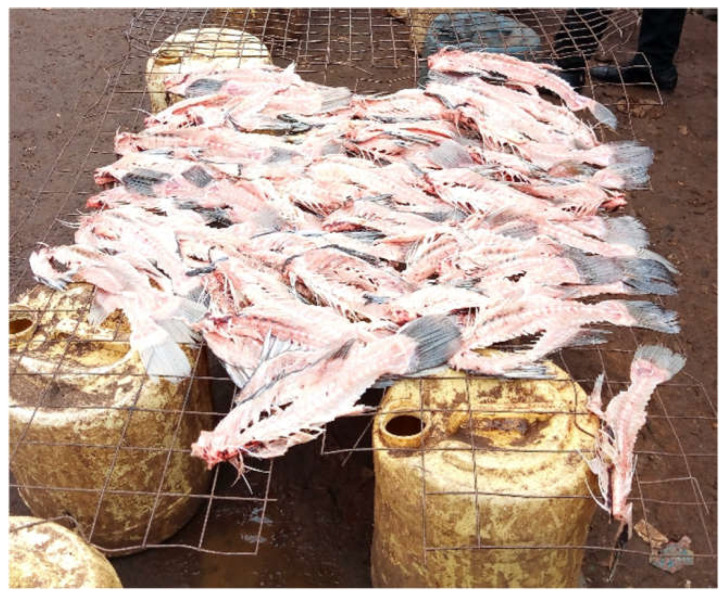
A photo of fish being sold in the informal settlements.

Beyond their own experiences, perceptions about quality were informed by advertisements and promotions. For instance, one woman noted, “*I usually see big fish with a lot of flesh in it being shown on TVs and I admired it*” (FGD1K), which contrasts with the fish sold in the informal settlements that is sold in parts (e.g., head, tail, etc.) with little flesh (see Figure 4).

## 4. Discussion

This study examined the food environments within two informal settlements in Nairobi, Kenya. Overall, we found the dimensions of the food environments in both informal settlements to be very similar and that the informal built food environment was the dominant source of food within the settlements. The most common foods sold by vendors were fruits and vegetables; however, there was limited diversity of fruits available. Animal-source foods were considered relatively expensive as compared to plant-based foods, including prepared fried snacks, and women perceived their quality to be low. We found that the way women interacted with their food environments was influenced by individual factors such as income, time, and preferences. Our study is a snapshot of the multiple dimensions of food environments in informal settlements, with the potential to inform the design of interventions aimed at supporting healthy food choices. Moreover, it provides insight into the use of mixed methods to comprehensively describe food environments, and consumers’ interaction with them, in informal settlements.

Although much of the food environment literature in high-income countries has focused on the mapping of fast-food chains and corner and grocery stores to measure food access, the types of food access points in LMICs are often dominated by a variety of small kiosks and street vendors within the informal built environment. If these types of food outlets are not captured in food environment mapping exercises, much of the foods that people living within these communities access will not be captured. Even in New York City, about a fifth of all food access points would be missed if street vendors and storefront businesses not primarily focused on selling food were not included in food environment mapping [34]. This phenomenon is likely even more pronounced in LMICs. We found that the highest proportion of food outlets in both informal settlements were selling fresh produce, which has also been found in previous studies [35]. Focus group participants indicated that they would purchase specific foods from different types of food outlets based on differentials in terms of either cost (e.g., fruit and vegetables cheaper at wet markets), quality (e.g., staple foods less likely to be expired at the supermarket vs. kiosks), or convenience (e.g., kiosks closer than wet markets, cooking time, etc.), which is aligned with other findings of purchasing patterns in Kenya [17]. Our previous work characterized these trade-offs that women make when making food purchasing decisions in informal settlements in Kenya [31].

We found that fresh produce of fairly high quality was available within the informal settlements, and we also found a high availability of fried foods such as french fries and mandazi (fried dough) as well as prepared meals being sold by street vendors (e.g., githeri). In contrast to other studies examining food environments in sub-Saharan Africa [18], commercially processed foods were not as prevalent in our study settings. Much of the global discussion around poor quality diets stemming from the nutrition transition has centered on an increase in ultra-processed foods. However, in informal settlements such as the ones examined in Nairobi, most of the foods sold are unpackaged and unbranded and do not technically meet the definition of ultra-processed foods based on the NOVA classification [35]. Thus, using packaged or ultra-processed foods as the only (or main) proxy for diet quality may misrepresent the true quality of the foods being consumed. Moreover, these classifications do not adequately address the quality of the oil used for many of these foods (e.g., food sold by street vendors), which was identified as a concern in this study and has been found to be poor in some LMICs [36,37].

Although ultra-processed foods were not a predominant part of the food environments examined in this study, participants reported seeing promotional advertisements for them, including in movies and television. These foods were still considered prohibitively expensive, but as incomes within these populations increase, there may be a shift towards higher consumption of these foods. This is problematic given that the burden of overweight/obesity and diet-related NCDs among adult women is already high in LMICs [38,39]. Moving forward in these communities, social marketing campaigns that highlight the importance of limiting consumption of energy-dense, nutrient-poor packaged and street foods might be necessary, while concomitantly increasing consumption of nutrient-rich foods. Although we found high levels of nutritional knowledge in our previous work in these settings [31], much of the focus has been on the components of a healthy diet with less of a focus on foods that should be limited in consumption.

There were many individual factors that influenced the way in which women described their ability to interact with their food environments. Affordability was a key driver of food choice in these settings, leading to trade-offs between the price of food and its quality, convenience, etc. [31]. Our focus group participants reported convenience, time, price, and quality trade-offs based on where they purchased foods (e.g., supermarket vs. kiosk). Although we found that the quality of produce in the informal settlements was generally rated as relatively high, focus group participants indicated that the quality at the wet markets that were adjacent to the settlements were higher than those sold in the kiosks within the informal settlements. This differential in terms of quality has also been seen in high-income settings where the quality of produce in low-income neighborhoods is poorer than in higher-income neighborhoods [40,41]. However, accessing the wet markets, given the distance from the settlements and poor road conditions in the rainy season, was considered less convenient than purchasing foods from kiosks sold in the informal settlements. Convenience was also a key factor that influenced women’s purchasing of foods from street vendors. More specifically, the convenience of not having to prepare food (including cleaning up after cooking), coupled with the perceived cost-savings related to not using as much cooking fuel, led women to purchase more prepared foods from street vendors. Given that people living in these settlements will likely continue to face time constraints, improving the quality of street food available is important. There have been programs in other countries to improve the food safety [42] as well as the ingredients used in foods prepared by street vendors [43] and a similar approach might be possible in Nairobi. Moreover, there is a need for the development and promotion of culturally relevant, low-cost, nutritious processed foods that take less time and cooking fuel to prepare in order to improve convenience and reduce costs. For example, processed beans that take less time and less cooking fuel to cook.

We used a variety of methods to capture the different dimensions of the food environment and the way that women interface with it. Although there was value in capturing both objective and perceived data across the different dimensions of the food environment, we identified gaps in the information that some of these tools provide. For example, women in our study mentioned aspects of quality that go beyond what the ProDesirability tool can capture. Concerns related to food adulteration appeared much more prominent than other aspects of food quality. This highlights the need to develop new tools that can provide additional information about food quality and safety across a greater diversity of foods to better inform our understanding of how this food environment dimension influences food choice. Another clear gap in the methods relates to measuring the convenience dimension of food environments. Although we were able to collect information about “convenience foods”, convenience goes beyond just the type of food. Future work should focus on developing easy to use tools that are able to capture the aspects of food environment dimensions that truly influence food choice.

### Limitations

Although our study has many strengths, it also has limitations. We were limited in our inability to map all of the villages in each of the informal settlements given the large number of vendors in each of the informal settlements. Although we selected villages based on feedback from our community partners, these boundaries did not encompass the totality of vendors and markets that women accessed to purchase food. We were also unable to capture photos of the majority of vendors, which influenced our ability to conduct a complete analysis of the foods sold in restaurants. Given that the food environments in these settings are dynamic, as noted by our participants, there are likely significant changes based on season. Although we would have ideally captured external food environment data in two different seasons, our focus group discussion data illuminated some of the challenges perceived by participants in accessing and affording different foods across seasons. Moreover, we captured food price data based on the units typically sold. However, this creates challenges in terms of comparing across food groups. Lastly, our qualitative data collection focused exclusively on women although other members of the households (men, adolescents, etc.) make decisions around food purchasing as well. Our future work will aim to further address these limitations.

## 5. Conclusions

We found the food environments in two informal settlements in Kenya to be very similar and to comprise predominantly of informal food vendors/outlets. Although nutritious foods were available within the settlements, animal-source foods and fruit in particular were relatively expensive. To support healthy diets in the food environments in which people interact with in informal settlements in Nairobi, Kenya, interventions targeting both the external food environment and individual factors are necessary. Increasing the diversity of fruits available in the informal settlements, improving the quality of street food, reducing the cost of animal-source foods, etc. could improve the ability of consumers to access more nutritious foods. Alongside these approaches, social marketing campaigns or behavior change communication strategies should highlight the importance of limiting consumption of energy-dense, nutrient-poor foods in order to attenuate the growing dual burden of malnutrition in these settings.

## Figures and Tables

**Table 1:** A summary of the ProColor diversity scores of vegetables and fruits in villages within Kibera and Mukuru.

Informal Settlement	Village	Dark Leafy Greens (#)	Types	Green (#)	Types	Red (#)	Types	Orange and Yellow (#)	Types	Purple and Blue (#)	Types	White (#)	Types
Vegetable Diversity													
Kibera	Gatwekera	8	Collard greens, romaine lettuce, amaranth, nightshade, chard, spinach, scallions, kale	4	Green pepper, avocado, peas, cucumber, lettuce	2	Red pepper, tomato	1	Sweet potato, corn, squash, carrot, yellow zucchini, yam		Purple butternut squash	4	Garlic, onion, green onion, ginger, eggplant, potato
	Kianda	8		5		1		3				6	
	Olympic	7		5		2		4		1		4	
	Soweto West	7		3		2		1				5	
Mukuru	Railway	7	Collard greens, romaine lettuce, amaranth, nightshade, chard, spinach, scallions, kale	3	Green pepper, avocado, peas, lettuce	1	Red pepper, tomato	2	Corn, squash, carrot, yellow zucchini		Cabbage	3	Garlic, onion, green onion, ginger, eggplant, potato, mushroom
	Sisal	6		4		2		2				4	
	Vietnam	6		3		1		3				7	
	Wape Wape	7		3		2		2		1		6	
**Fruit Diversity**													
Kibera	Gatwekera			1	Lime		Watermelon	2	Mango, orange, papaya, pineapple, lemon		Blueberry	2	Coconut, banana, apple
	Kianda			1		1		4		1		3	
	Olympic			1		1		5				3	
	Soweto West			1				2				1	
Mukuru	Railway			1	Lime, honeydew	1	Watermelon	3	Mango, papaya, pineapple, lemon			1	Coconut, banana
	Sisal			1				3				1	
	Vietnam			2		1		4				1	
	Wape Wape			1				2				2	

#: Number of vegetables or fruits of that color available within the village

## Data Availability

Data will be made available upon request to corresponding author.

## Supplementary Materials

The following supporting information can be downloaded at: https://www.mdpi.com/article/10.3390/nu14132571/s1, Figure S1: An overview of the types of vendors in Kibera (panel A) and Mukuru (panel B), Figure S2: An overview of the summary of price observations for each food in both Kibera and Mukuru, Table S1: An overview of the types of vendors selling food in the informal settlements, Table S2: Inter-rater reliability of ProDesirability ratings of produce quality, Table S3: The average price of key food items in Kibera and Mukuru, Table S4: An overview of the sensory properties of fruits and vegetables in Kibera and Mukuru.
