# Supplementary material for: Food Environments and Their Influence on Food Choices: A Case Study in Informal Settlements in Nairobi, Kenya
Source: Nutrients. 2022 Jun 21;14(13):2571. doi: 10.3390/nu14132571 (PMC9268418; doi:10.3390/nu14132571)
Supplement: Supplementary file 1 [file nutrients-14-02571-s001.zip › nutrients-1766201-supplementary.pdf]

Supplementary Materials

Table S1. An overview of the types of vendors selling food in the informal settlements.

Vendor type	Description	Photo examples
Butcher	Food stall selling primarily meat. Some butchers may also sell roasted meat or fresh produce/eggs	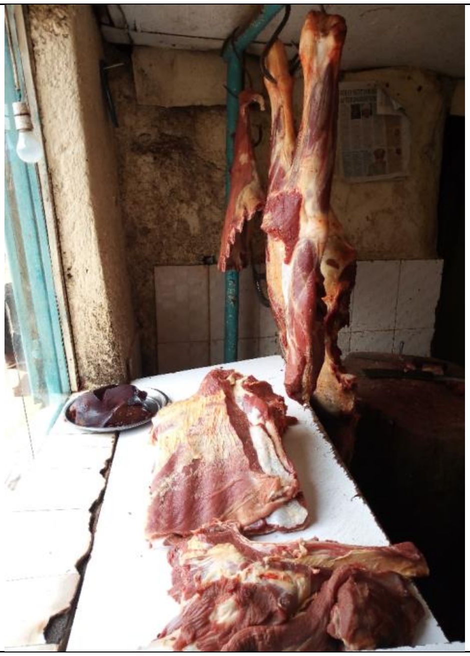

Kiosk	Food stalls selling ready-to-eat meals, fresh produce, fish, eggs, sweets & chocolate or grains and legumes. There is some permanent infrastructure. In some cases, there may also be seating (e.g., kiosks selling ready-to-eat foods and tea also known as kibandas)	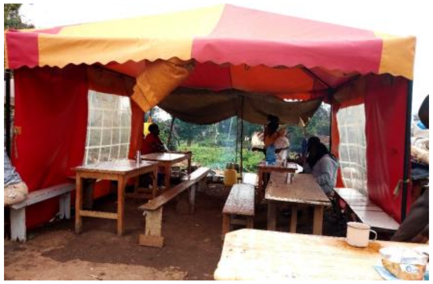 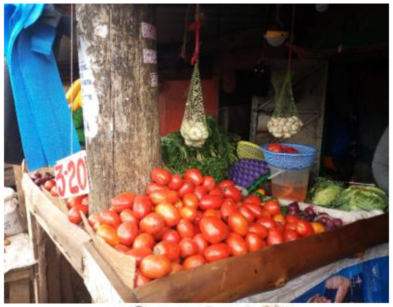 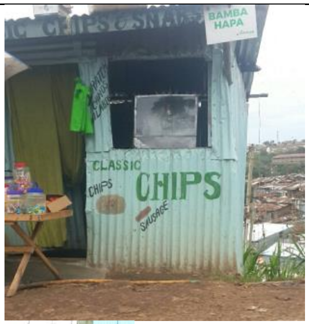 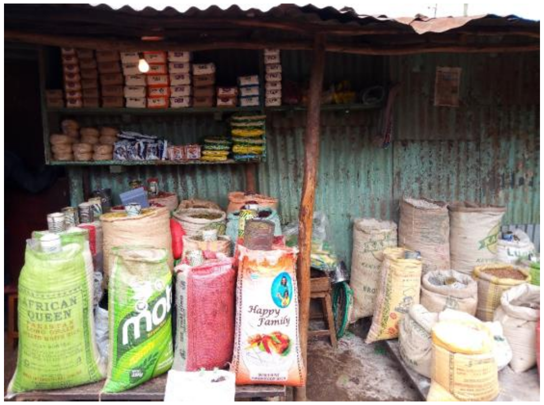 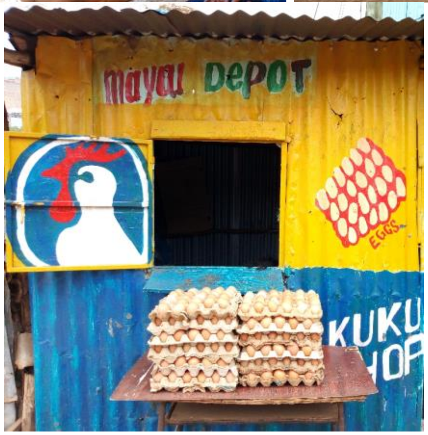

Restaurants	Small independently owned restaurants selling meals, beverages and snacks. Typically referred to as hotels in the informal settlements.	(photographs below)

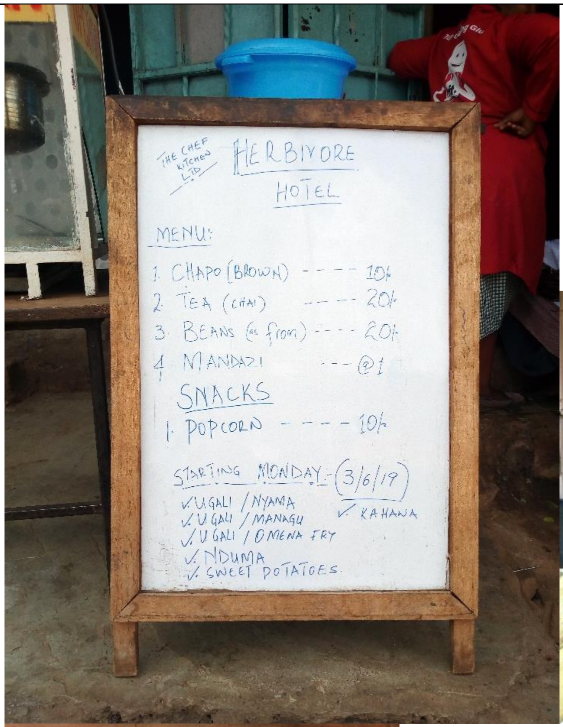

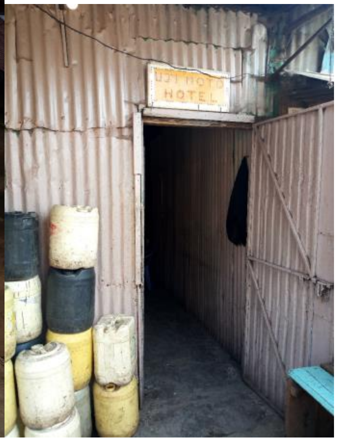

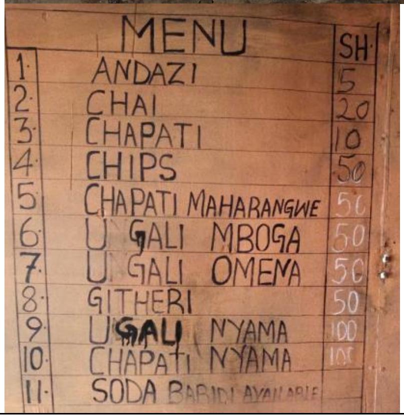

Small grocer	Mom and pop shop selling a variety of food and non-food items. They typically offer a variety of processed and ultra-processed foods. Some also sell fresh produce, eggs and/or milk. These are smaller than a supermarket but larger than a kiosk. Often referred to as Dukas.	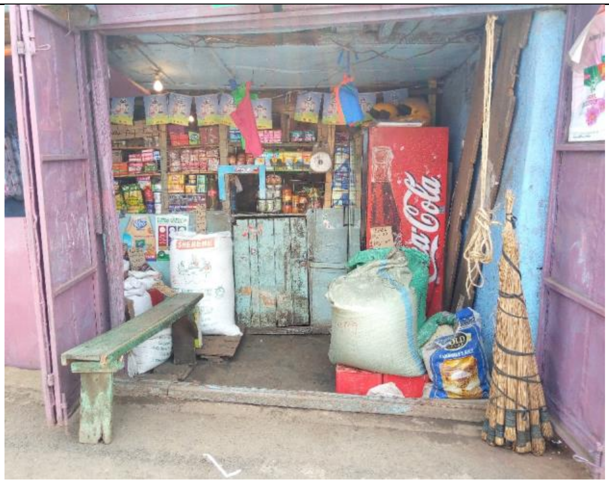
Wholesaler	Independently owned shops that sell packaged food and non-food items in relatively large quantities primarily to retailers rather than direct to consumers.	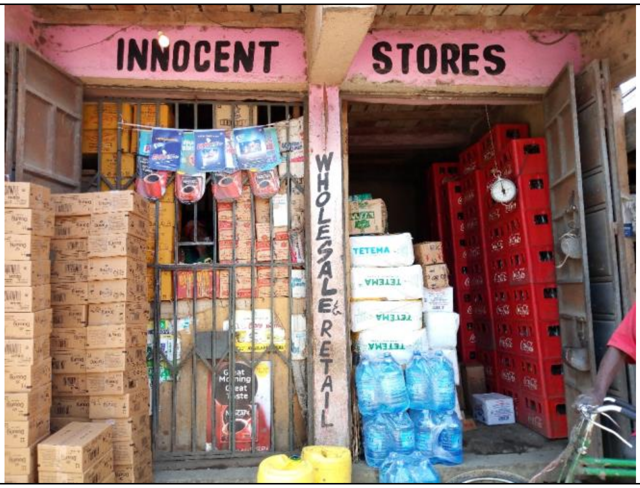

Milk vending machine	Milk dispensing or vending machine (referred to locally as milk ATMs) selling milk and in some cases other dairy products (e.g., ice cream)	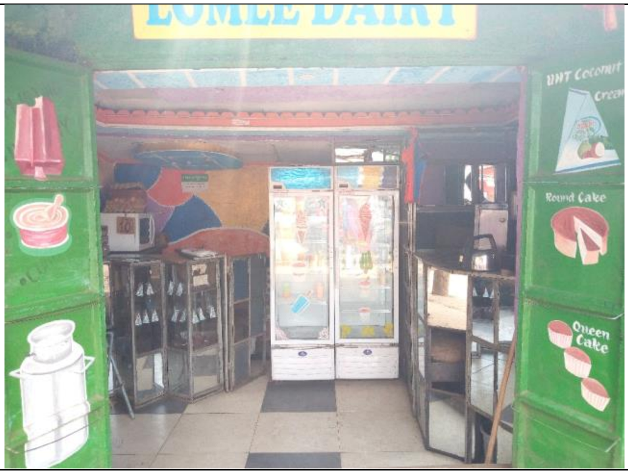

Street vendors	Small mobile or temporary roadside stands selling a variety of foods including fresh produce, fish, sweets and chocolate, ready-to-eat foods, etc. Typically there is limited infrastructure. Most vendors sell their foods at a fixed location.	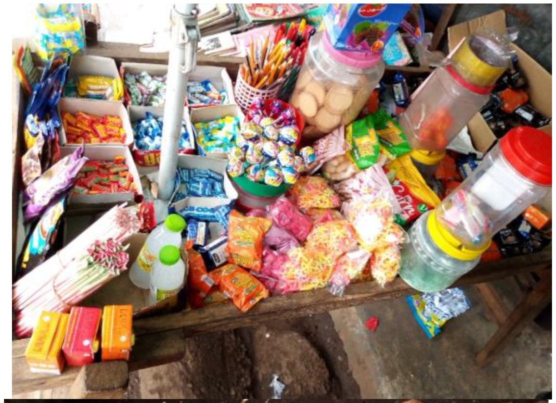 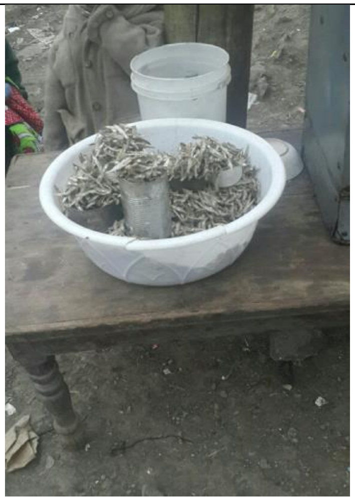 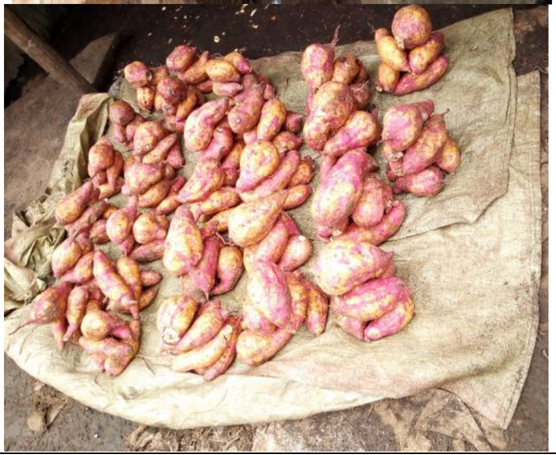
Small supermarket*	Fixed structures selling a variety of processed, ultra-processed and fresh foods as well as non-food items. Larger than the small grocers but still relatively small in size. Supermarkets	

	in the informal settlements were independently owned.	

*Note: No photos of supermarkets were taken

Table S2. Inter-rater reliability of Pro-Desirability ratings of produce quality

Location	Statistical test	Overall desirability	Touch and firmness	Visual appeal	Size	Aroma
Kibera	Correlation coefficient	0.734	0.746	0.759	0.736	0.78
	P-value	<0.001	<0.001	<0.001	<0.001	<0.001
	Kibera (kappa ± SE)	0.244 ± 0.078	0.423 ± 0.055	0.336 ± 0.051	0.434 ± 0.051	0.437 ± 0.054
	P-value	<0.001	<0.001	<0.001	<0.001	<0.001
Mukuru	Correlation coefficient	0.664	0.562	0.493	0.597	0.634
	P-value	<0.001	<0.001	<0.001	<0.001	<0.001
	Mukuru (kappa ± SE)	0.418 ± 0.051	0.291 ± 0.052	0.248 ± 0.051	0.319 ± 0.051	0.328 ± 0.051
	P-value	<0.001	<0.001	<0.001	<0.001	<0.001

Table S3. The average price of key food items in Kibera and Mukuru

**Food groups**	**Food items**	**Units of price measurement**	**Mukuru**		**Kibera**	
			**Price per unit (Kenyan Shillings)**		**Price per unit (Kenyan Shillings)**	
			Average ($KES)	Standard Deviation	Average ($KES)	Standard Deviation
Grains, white roots and tubers and plantains	Bread	Loaf	49	6	50	0.0
	Maize flour	Per kilogram	118	10	60	1.1
	Rice	Per kilogram	90	9	98	6.6
Pulses (beans, peas and lentils)	Nyayo beans	Per kilogram	89	4	98	8.9
	Rose coco beans	Per kilogram	104	8	108	11.2
Dairy	Milk	Per 500 milliliters	45	9	50	3.7
Meat, poultry and fish	Beef	Per kilogram	402	9	395	36.9
	Chicken	Per piece	230		370	114.9
	Nile perch	Per piece	75	35	87	70.9
	Omena	Per cup	25	7	43	15.9
	Tilapia	Per piece	43	10	153	38.5
Eggs	Egg	Per egg	10	0	10	0.4
Dark green leafy vegetables	Kale	Per bundle	5	3	5	1.1
Other vitamin A-rich fruits and vegetables	Mango	Per piece	6	3	13	7.5
	Papaya	Per piece	180		28	17.2
	Sweet potatoes	Per piece	68	11	68	10.6
Other vegetables	Cabbage	Per piece	76	43	88	26.2
	Onion	Per piece	5	0	6	1.8
	Tomato	Per piece	6	2	8	2.6

Other fruits	Avocados	Per piece	11	6	22	9.1
	Banana	Per piece	5	0	8	2.5
	Coconut	Per piece	40		40	
	Orange	Per piece	6	2	9	5.4
	Pineapple	Per slice	18	3	18	2.9
	Watermelon	Per piece	89	110	19	6.9
Deep fried foods	Bhajia	Per plate	24	14	24	14.3
	French fries	Per plate	19	4	38	13.1
	Mandazi	Per piece	5	0	5	1.1
Beverages	Soda	Per 500 milliliters	50	0	50	0.0
	Tea	Per cup	17	5	17	4.9
Other prepared snacks and meals	Chapati	Per piece	10	2	11	1.8
	Rice and beans	Per plate	38	15	53	25.7
	Smokies	Per piece	25	0	25	0.0
	Ugali	Per plate	33	6	33	5.8
	Githeri	Per plate	18	9	18	14.6
Other staples	Sugar	Per kilogram	109	19	113	7.7
	Cooking oil	Per Litre	143	19	123	5.8

Table S4. An overview of the sensory properties of fruits and vegetables in Kibera and Mukuru

		**Overall desirability**		**Aroma**		**Size**		**Touch/firmness**		**Visual appeal**	
**Fruits**		Kibera (mean ± SD)	Mukuru (mean ± SD)	Kibera (mean ± SD)	Mukuru (mean ± SD)	Kibera (mean ± SD)	Mukuru (mean ± SD)	Kibera (mean ± SD)	Mukuru (mean ± SD)	Kibera (mean ± SD)	Mukuru (mean ± SD)
	Banana	4.92 ± 0.96	5.21 ± 0.85	5.38 ± 1.00	5.38 ± 1.00	5.17 ± 1.06	5.17 ± 1.06	4.98 ± 1.10	4.98 ± 1.10	4.83 ± 1.04	4.83 ± 1.04
	Mango	4.41 ± 0.87	4.44 ± 0.85	5.20 ± 0.63	5.20 ± 0.63	4.75 ± 0.81	4.75 ± 0.81	4.86 ± 0.77	4.86 ± 0.77	4.45 ± 0.90	4.45 ± 0.90
	Orange	5.27 ± 0.64	4.83 ± 0.75	5.60 ± 0.54	5.60 ± 0.54	5.13 ± 0.76	5.13 ± 0.76	5.58 ± 0.65	5.58 ± 0.65	5.42 ± 0.61	5.42 ± 0.61
	Pineapple	5.50 ± 0.52	4.90 ± 0.72	4.92 ± 1.16	4.92 ± 1.16	5.33 ± 0.65	5.33 ± 0.65	5.17 ± 0.94	5.17 ± 0.94	5.08 ± 0.67	5.08 ± 0.67
**Vegetables**	Cabbage	5.04 ± 1.15	5.02 ± 0.96	5.08 ± 1.11	5.08 ± 1.11	4.88 ± 1.14	4.88 ± 1.14	5.17 ± 1.15	5.17 ± 1.15	5.02 ± 1.16	5.02 ± 1.16
	Kale	5.08 ± 0.92	4.98 ± 1.12	5.17 ± 1.06	5.17 ± 1.06	5.00 ± 1.19	5.00 ± 1.19	5.04 ± 1.18	5.04 ± 1.18	5.25 ± 1.14	5.25 ± 1.14
	Onion	5.13 ± 0.85	5.13 ± 0.85	5.21 ± 0.94	5.21 ± 0.94	4.73 ± 1.07	4.73 ± 1.07	5.25 ± 0.84	5.25 ± 0.84	5.29 ± 0.82	5.29 ± 0.82
	Sweet potato	4.96 ± 1.08	4.96 ± 1.08	5.33 ± 0.82	5.33 ± 0.82	5.29 ± 1.00	5.29 ± 1.00	5.13 ± 1.03	5.13 ± 1.03	4.92 ± 1.25	4.92 ± 1.25
	Tomato	5.42 ± 0.87	4.58 ± 1.13	5.40 ± 0.84	5.40 ± 0.84	5.23 ± 0.90	5.23 ± 0.9	5.44 ± 1.03	5.44 ± 1.03	5.33 ± 1.00	5.33 ± 1.00

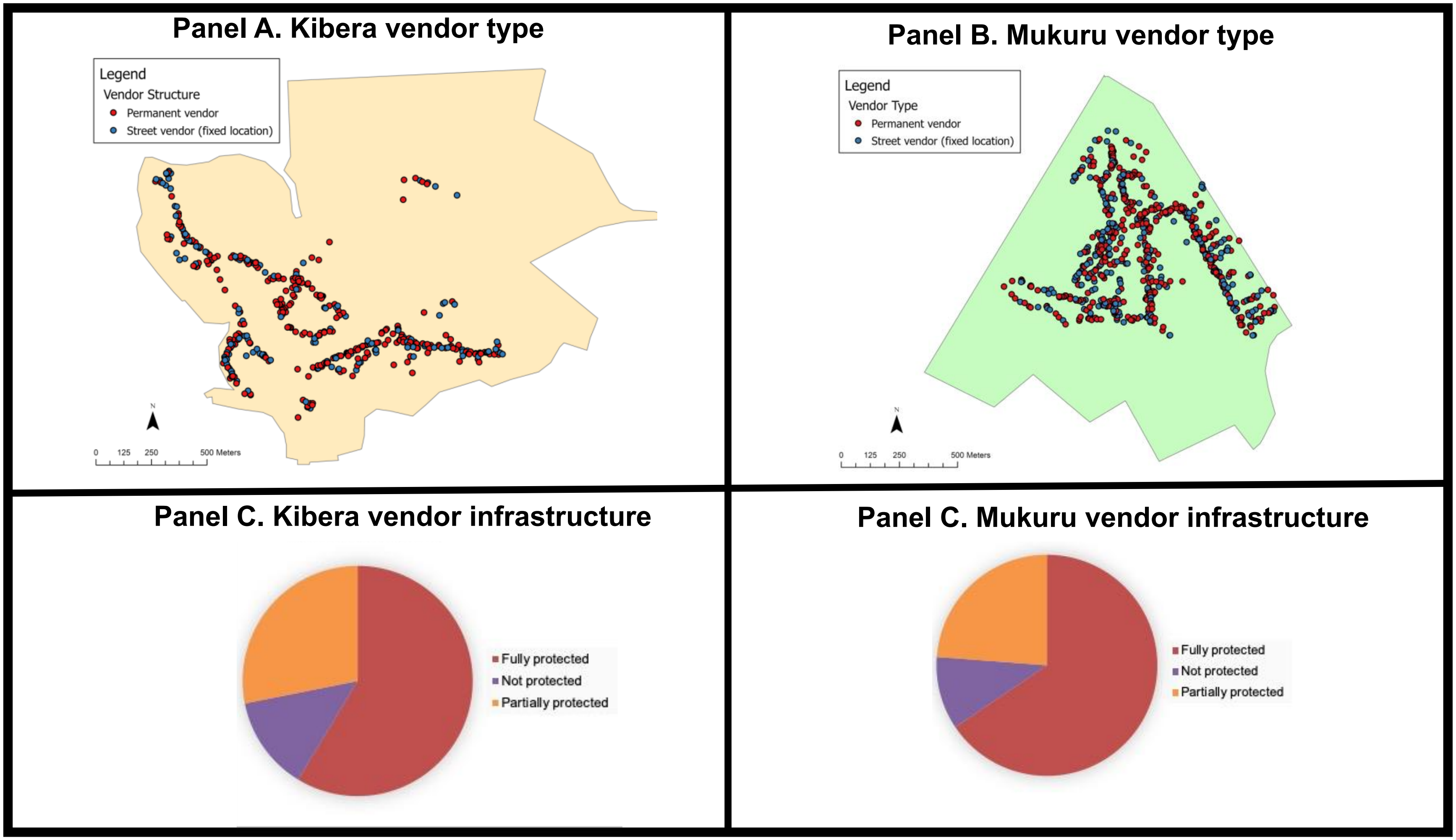

Figure S1. An overview of the types of vendors in Kibera (panel A) and Mukuru (panel B).

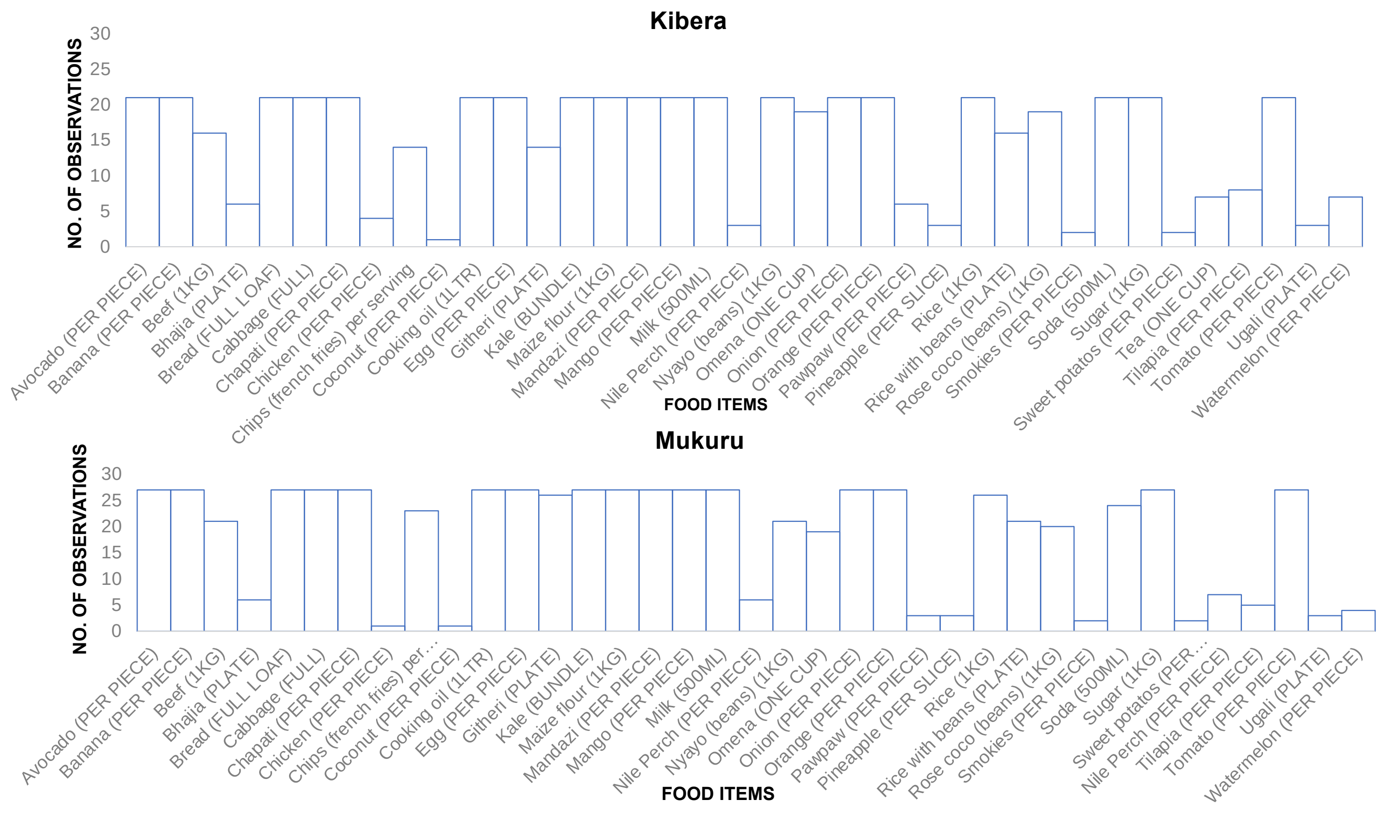

Figure S2. An overview of the summary of price observations for each food in both Kibera and Mukuru.
